# The role of intra-articular neuronal CCR2 receptors in knee joint pain associated with experimental osteoarthritis in mice

**DOI:** 10.1186/s13075-021-02486-y

**Published:** 2021-04-07

**Authors:** Shingo Ishihara, Alia M. Obeidat, David L. Wokosin, Dongjun Ren, Richard J. Miller, Anne-Marie Malfait, Rachel E. Miller

**Affiliations:** 1 Department of Internal Medicine, Division of Rheumatology, Rush University Medical Center, 1735 W Harrison St, Room 714, Chicago, IL 60612 USA; 2 Department of Physiology, Northwestern University, Chicago, IL 60611 USA; 3 Department of Pharmacology, Northwestern University, Chicago, IL 60611 USA

**Keywords:** Osteoarthritis, Hyperalgesia, Sensitization, Pain, CCR2, CCL2, Animal model

## Abstract

**Background:**

C–C chemokine receptor 2 (CCR2) signaling plays a key role in pain associated with experimental murine osteoarthritis (OA) after destabilization of the medial meniscus (DMM). Here, we aimed to assess if CCR2 expressed by intra-articular sensory neurons contributes to knee hyperalgesia in the early stages of the model.

**Methods:**

DMM surgery was performed in the right knee of 10-week-old male wild-type (WT), *Ccr2* null, or *Ccr2*^RFP^ C57BL/6 mice. Knee hyperalgesia was measured using a Pressure Application Measurement device. CCR2 receptor antagonist (CCR2RA) was injected systemically (i.p.) or intra-articularly (i.a.) at different times after DMM to test its ability to reverse knee hyperalgesia. In vivo Ca^2+^ imaging of the dorsal root ganglion (DRG) was performed to assess sensory neuron responses to CCL2 injected into the knee joint cavity. CCL2 protein in the knee was measured by ELISA. *Ccr2*^RFP^ mice and immunohistochemical staining for the pan-neuronal marker, protein gene product 9.5 (PGP9.5), or the sensory neuron marker, calcitonin gene-related peptide (CGRP), were used to visualize the location of CCR2 on intra-articular afferents.

**Results:**

WT, but not *Ccr2* null, mice displayed knee hyperalgesia 2–16 weeks after DMM. CCR2RA administered i.p. alleviated established hyperalgesia in WT mice 4 and 8 weeks after surgery. Intra-articular injection of CCL2 excited sensory neurons in the L4-DRG, as determined by in vivo calcium imaging; responses to CCL2 increased in mice 20 weeks after DMM. CCL2, but not vehicle, injected i.a. rapidly caused transient knee hyperalgesia in naïve WT, but not *Ccr2* null, mice. Intra-articular CCR2RA injection also alleviated established hyperalgesia in WT mice 4 and 7 weeks after surgery. CCL2 protein was elevated in the knees of both WT and *Ccr2* null mice 4 weeks after surgery. Co-expression of CCR2 and PGP9.5 as well as CCR2 and CGRP was observed in the lateral synovium of naïve mice; co-expression was also observed in the medial compartment of knees 8 weeks after DMM.

**Conclusions:**

The findings suggest that CCL2-CCR2 signaling locally in the joint contributes to knee hyperalgesia in experimental OA, and it is in part mediated through direct stimulation of CCR2 expressed by intra-articular sensory afferents.

**Supplementary Information:**

The online version contains supplementary material available at 10.1186/s13075-021-02486-y.

## Background

The C–C chemokine receptor type 2, CCR2, is a seven-transmembrane domain G-protein coupled receptor (GPCR) that acts as the receptor for the C–C motif chemokine ligands (CCL), CCL2, CCL7, and CCL12 [1]. CCL2-CCR2 signaling is potently chemotactic for monocytes and immune cells, and since CCR2 is a GPCR, it provides a pharmacologically tractable target [2, 3].

In the field of rheumatology, CCR2 has received a great deal of attention as a potential target especially for inflammatory arthritis, because of its well-documented pro-inflammatory and chemotactic actions [4]. CCR2 has also been explored as a target in osteoarthritis (OA) [5]. OA is the most common form of arthritis, affecting 27 million people in the USA [6]. The disease is characterized by progressive joint damage, associated with chronic pain and disability [7]. Disease-modifying OA drugs (DMOADs) that slow down or halt the progression of joint damage and at the same time manage symptoms, do not yet exist [8]. A broad range of analgesic therapies is available to patients, but the most commonly prescribed painkillers suffer from lack of efficacy and problems with side effects or addiction. Therefore, chronic OA pain is often inadequately controlled and constitutes a major health problem [9]. Hence, extensive efforts in academia and industry are focused on identifying safe new targets for OA pain [10, 11].

Increasingly, experimental evidence suggests that innate immunity and low-grade inflammation contribute to the pathogenesis of OA [12], providing a solid rationale for exploring CCR2 as a pharmacological target for this disease. Studies in rodent models of OA, however, that aimed to inhibit CCR2 signaling either through genetic ablation of *Ccr2* or through pharmacological blockade with a CCR2 receptor antagonist (CCR2RA) have been largely disappointing, showing no or limited protection from joint damage (reviewed in [5]). Yet, at the same time, studies in a mouse model where experimental OA is induced by surgical destabilization of the medial meniscus (DMM) revealed that CCR2 signaling may be essential for OA-associated pain [13–15]. Indeed, several laboratories have reported a substantial contribution of CCL2-CCR2 signaling to pain-related behaviors associated with progressive joint damage after DMM surgery, including persistent mechanical allodynia, locomotive deficits, and weightbearing deficits (reviewed in [5]).

Pain is sensed by specialized afferent neurons, called nociceptors, that innervate the skin and internal tissues, including joints [16]. Nociceptors are activated by noxious stimuli, such as heat, acid, or a painful mechanical stimulus [17] through an array of specialized receptor channels [18]. For example, transient receptor potential cation channel vanilloid 1 (TRPV1) is activated by noxious heat [19], acid-sensing ion channel (ASIC) is activated by acid [20], and Piezo-type mechanosensitive ion channel component 2 (PIEZO2) is activated by noxious mechanical stimuli [21]. Activation of these receptors results in the generation of action potentials, propagated by ion channels such as voltage-gated sodium channels like Na_V_1.8 and Na_V_1.7 [18]. These action potentials carry the painful signal along the axon to the cell bodies in the dorsal root ganglia (DRG), and onward to the dorsal horn of the spinal cord, where the first synapse occurs with second-order neurons in the central nervous system (CNS) [17]. In addition to these specialized channels, nociceptors also express receptors for ligands that are present as part of an inflammatory response, such as receptors for cytokines, chemokines, nerve growth factor (NGF) [18] and Toll-like receptors (TLRs) [22, 23]. Thus, many mediators present in the inflammatory milieu can have excitatory effects on nociceptive neurons. This has been well documented for CCL2 (also known as monocyte chemoattractant protein 1 or MCP-1) which, upon binding of CCR2 expressed by DRG neurons, causes rapid excitation through the transactivation of TRPV1 and other ion channels [24]. Concordantly, CCL2 elicits rapid pain behaviors when injected into the paw [24]. In addition to these direct neuronal effects, CCR2 signaling may contribute to painful responses in the context of tissue injury through recruiting macrophages and other immune cells to the injured site, and these cells in turn become local sources of pro-inflammatory, algogenic molecules, further establishing a painful vicious cycle [25].

In the context of OA, studies in the DMM model have revealed that protection from persistent pain in *Ccr2* null mice is associated with reduced infiltration by macrophages in the DRGs [13]. We also showed that cell bodies of sensory neurons in knee-innervating L4-DRGs have increased expression of CCR2 after DMM surgery [13]. However, to date, it is not known if CCR2 is expressed on afferents that are located intra-articularly in joint tissues and if direct activation of these receptors by locally produced CCL2 might contribute to joint pain. Here, we set out to address this question using healthy naïve mice and mice with experimental OA induced by DMM surgery.

## Methods

### Animals

For these studies, a total of 170 mice were used. All animal experiments were approved by the Institutional Animal Care and Use Committee at Rush University Medical Center. Animals were housed with food and water ad libitum and kept on 12-h light cycles. Wild type (WT) C57BL/6 J male mice were bred at Rush. *Ccr2* null mice on a C57BL/6 J background were obtained from Taconic Farms (#3736). Pirt-GCaMP3 mice were received as a gift from Dr. Xinzhong Dong and bred at Rush. These knock-in mice express the fluorescent calcium indicator GCaMP3 in ~ 90% of all DRG sensory neurons, and not in other peripheral or central tissues, through the Pirt promoter [26]. *Ccr2* null × Pirt-GCaMP3 mice were generated through two back-crosses at Rush. *Ccr2*^RFP^ mice were obtained from the Jackson Laboratory (#017586).

### Surgery

DMM surgery was performed in the right knee of 10-week old male WT or *Ccr2* null mice as previously described [13, 27]. In brief, after medial parapatellar arthrotomy, the anterior fat pad was dissected to expose the anterior medial meniscotibial ligament, which was severed. The knee was flushed with saline and the incision closed. Sham surgery was identical to DMM except that the medial meniscotibial ligament remained intact.

### Knee hyperalgesia

Knee hyperalgesia was measured using a Pressure Application Measurement (PAM) device (Ugo Basile, Varese, Italy), as previously described [28]. Briefly, mice were restrained by the hand and the hind paw was lightly pinned to make the correct flexion at a similar angle for each mouse. The PAM transducer was pressed against the medial side of the knee and pressure applied against the knee. PAM software guided the user to apply a constantly increasing force (30 g/s) up to a maximum of 450 g. If the mouse tried to withdraw its knee, the force at which this occurred was recorded. Two measurements were taken and recorded per knee and the withdrawal force data were averaged. Knee hyperalgesia was assessed before surgery and 2, 4, 8, 12, and 16 weeks after DMM surgery in WT (*n* = 5) and in *Ccr2* null (*n* = 5) mice by an experimenter blinded to the mouse strain.

### Effect of systemically delivered CCR2RA on knee hyperalgesia

DMM surgery was performed in 10-week old male WT mice. Four, 8, 12, or 16 weeks after DMM surgery, mice received an intraperitoneal (i.p.) injection of CCR2 Receptor Antagonist (CCR2RA, RS 504393, Tocris, 5 mg/kg) (*n* = 5 mice/time point) or vehicle control (100% DMSO_4_) (*n* = 4 mice/time-point). Knee hyperalgesia was assessed right before injection, and again 1, 2, and 3 h after injection, by an experimenter blinded to the groups.

### Effect of locally delivered CCL2 on knee hyperalgesia

An independent set of 10-week old naïve WT and *Ccr2* null mice were injected i.a. with either CCL2 (500 ng in 0.1% BSA suspended in 5 μL PBS) (479-JE-010/CF, R&D Systems) (*n* = 11 WT and *n* = 5 *Ccr2* null mice) or vehicle (0.1% BSA suspended in 5 μL PBS) (*n* = 11 WT and *n* = 4 *Ccr2* null mice). Knee hyperalgesia was assessed right before injection, and 1, 2, and 4 h after injection, and again 24 h after injection by an experimenter blinded to the groups.

### Effect of locally delivered CCR2RA on knee hyperalgesia

DMM surgery was performed in 10-week old male WT mice, and 4 and 7 weeks later, CCR2RA (BMS CCR2 22, R&D Systems) was injected intra-articularly (i.a.) (0.5 mg/kg in 50% ethanol) (*n* = 4 at 4 weeks, *n* = 6 at 7 weeks) or vehicle (50% ethanol) (*n* = 4 at 4wk, *n* = 6 at 7 weeks) using independent set of WT mice after DMM surgery. The injected volume was 5 μL (target concentration: 0.5 mg/kg body weight). Knee hyperalgesia was assessed right before injection, and again 1, 2, and 4 h after injection, by an experimenter blinded to the mouse strain.

### CCL2 protein levels in whole knee joint extracts

Sham or DMM surgery was performed in 10-week old male WT or *Ccr2* null mice. Naïve WT mice were also used. Four or 8 weeks after surgery, ipsilateral knee joints were collected and flash frozen. The entire knee joint was collected, including femoral condyles and tibial plateaux, with the knee joint kept intact; attached tissues (skin, muscle) were removed. Protein extraction was achieved by grinding the knee joint under liquid nitrogen, incubating the tissue in lysis buffer with protease inhibitors overnight at 4 °C, and reserving the supernatants. CCL2 protein levels were measured by ELISA (R&D system) and normalized to total protein (bicinchoninic acid (BCA) assay, Thermo Scientific). (*n* = 4 mice naïve WT 14 weeks old; *n* = 6 WT sham + 4; *n* = 11 WT DMM + 4; *n* = 4 naïve WT 18 weeks old; *n* = 5 WT sham+ 8; *n* = 11 WT DMM + 8; *n* = 6 *Ccr2* null naïve 10 weeks old; *n* = 8 *Ccr2* null DMM + 4; *n* = 5 *Ccr2* null DMM + 8).

### In vivo calcium imaging

In study 1, naïve adult male mice, age 14–26 weeks (*n* = 8 Pirt-GCaMP3^+/−^; *n* = 8 *Ccr2* null^−/−^ × Pirt-GCaMP3^+/−^) were used. In study 2, adult male Na_V_1.8 cre × GCaMP6s loxp mice were used (naïve mice, age 21 weeks (*n* = 6) and DMM mice 20 weeks after surgery (*n* = 8)). All mice were deeply anesthetized using isoflurane (1.5–2% in O_2_), a laminectomy from vertebrae L2–L6 was performed, and the right-side L4 dorsal root ganglion (DRG) was exposed [29]. This DRG contains most of the sensory neurons that innervate the mouse knee joint [30, 31]. Silicone elastomer (World Precision Instruments) was used to cover the exposed DRG and surrounding tissue to avoid drying [32]. The mouse was positioned under a Prairie Systems Ultima In Vivo two photon microscope on a custom stage, using Narishige spinal clamps to slightly elevate the mouse in order to avoid motion artifacts associated with breathing. A Coherent Chameleon-Ultra2 Ti:Sapphire laser was tuned to 920 nm, and GCaMP3 or GCaMP6 signal was collected by using a bandpass filter for the green channel (490 to 560 nm). Image acquisition was controlled using PrairieView software version 5.3. Images of the L4 DRG were acquired at 0.7 Hz, with a dwell time of 4 μs/pixel (pixel size 1.92 × 1.92 μm^2^), and a 10× air lens (Olympus UPLFLN U Plan Fluorite, 0.3 NA, 10 mm working distance). The scanned sample region was 981.36 × 981.36 μm^2^. Anesthesia was maintained using isoflurane (1.5–2%) during imaging. For each mouse, a 30 G needle connected to a Hamilton syringe was inserted into the intra-articular space of the knee joint, imaging was started, 7 μL vehicle (0.1% BSA in PBS) was injected from frame 20–25, imaging continued until frame 100, and the needle was removed. After a 5-min recovery period, the process was repeated: a 30 G needle connected to a Hamilton syringe was inserted into the intra-articular space of the knee joint, imaging was started, 7 μL recombinant CCL2 (700 ng) was injected from frame 20–25, imaging continued until frame 100, and the needle was removed. Pilot experiments confirmed that repeated injections of saline did not induce responses. In addition, as a positive control, neuronal responses to a 200 g force applied to the ipsilateral hind paw were confirmed [29] prior to proceeding with the rest of the experiment. For each mouse, changes in [Ca^2+^]_i_ were quantified using a custom ImageJ macro to calculate the change in fluorescence in each frame t of a time series using the formula: ∆F/Fo, where Fo is the average intensity of the baseline period (15 frames) prior to injection [29]. Sensory neuron responses to either vehicle or to CCL2 were identified as cells having peak ∆F/Fo values during or after the application period that was greater than 4 times the standard deviation of the baseline period [32]. The total number of neurons imaged for each DRG was estimated by counting the number of neurons within a region of average density and extrapolating to the total imaged area (mean ± SEM: Pirt-GCaMP3 = 280 ± 36 neurons; *Ccr2* null × Pirt-GCaMP3 = 278 ± 17 neurons; naïve Na_V_1.8-GCaMP6s = 321 ± 38 neurons; DMM Na_V_1.8-GCaMP6s = 323 ± 33 neurons). The percentage of responses to either vehicle or to CCL2 was calculated using the formula: # responses / # total neurons imaged × 100.

### Knee immunohistochemistry

Ten-week-old male naïve *Ccr2*^RFP^ mice (*n* = 7) or *Ccr2*^*RFP*^ mice 8 weeks after DMM surgery (*n* = 3) were perfused transcardially with phosphate-buffered saline (PBS) followed by 4% paraformaldehyde (PFA) in PBS. The right knees were collected, post-fixed in PFA, decalcified in 14% EDTA and cryo-preserved in 30% sucrose. Twenty-μm-thick coronal sections were cut throughout the whole joint using the cryostat. Mid-joint sections were collected as previously described [33]. The mid-joint region comprises about 400-μm-thick region (20 sections) and was defined as the area where the meniscal horn is devoid of any ligament attachments. These sections were immunostained for protein gene product 9.5 (PGP9.5), a pan neuronal marker (rabbit polyclonal antibody, SAB4503057, Sigma) (*n* = 4 naïve mice), or for calcitonin gene-related peptide (CGRP) (rabbit polyclonal antibody, 24112, ImmunoStar), a marker for a subset of nociceptors (*n* = 3 naïve and *n* = 3 DMM + 8 weeks mice) [34–36]. Knee sections were blocked with 5% goat serum and 0.1% triton X-100 in PBS for 1 h at room temperature, then incubated overnight with primary antibodies against PGP9.5 (1:100) or CGRP (1:200) at 4 °C, followed by a secondary antibody, goat anti-rabbit conjugated Alexa Fluor 633 (Molecular Probes; 1:500). Sections were then imaged using a laser-scanning confocal microscope (Olympus IX70) with independent lasers used to excite the red and far red channels. Images were processed using Fluoview software (FV10-ASW 4.2 Viewer). All images were treated the same in terms of adjustments to brightness and contrast to minimize bias. Channels observed in the subchondral bone of the medial femoral condyles and tibial plateaux that contained CGRP+ and CCR2+/CGRP+ nerves were quantified as previously described [33]: two mid-joint sections (80 μm apart) per knee were used to count CGRP+ and CCR2+/CGRP+ channels; counts were averaged for each knee and compared with naïve mice.

### Statistical analysis

For knee hyperalgesia data, a one-way ANOVA with Bonferroni post-test was used to compare time points after DMM surgery in WT or *Ccr2* null mice to time 0. For knee hyperalgesia experiments testing injection of CCR2-RA or injection of CCL2, a repeated measures two-way ANOVA with Bonferroni post-test was applied to compare mice treated with vehicle to mice treated with CCR2RA or CCL2 at each time point. For in vivo calcium imaging, the number of sensory neuron responses to CCL2 was compared to the number of responses to vehicle for each strain of mice by paired two-tailed *t* test. In addition, for Na_V_1.8-GCaMP6s mice, the number of sensory neuron responses to CCL2 was compared between naïve and DMM mice by unpaired two-tailed *t* test. For knee protein extracts, data were analyzed by one-way ANOVA with Tukey post-test to compare treatment groups at each time point. For immunohistochemical analysis of osteochondral channels, the number of channels with nerves was compared between naïve and DMM mice by unpaired two-tailed *t* test. Statistical analyses were done using GraphPad Prism version 8.2.1 for Mac (GraphPad Software, San Diego, CA). Results are presented as mean ± SEM.

## Results

### Wild type mice but not *Ccr2* null mice develop primary knee hyperalgesia after DMM surgery

**Fig. 1:**
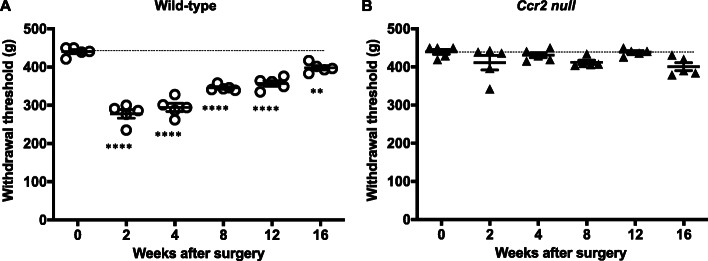
Knee hyperalgesia after DMM surgery. **a** WT (*n* = 5) mice and **b** *Ccr2* null mice (*n* = 5). The withdrawal threshold baseline is indicated by the dashed line. **p < 0.01, ****p < 0.0001 vs time 0. One-way ANOVA with Bonferroni’s multiple comparisons test to compare each time point to its respective time 0. Mean ± SEM

**Fig. 2:**
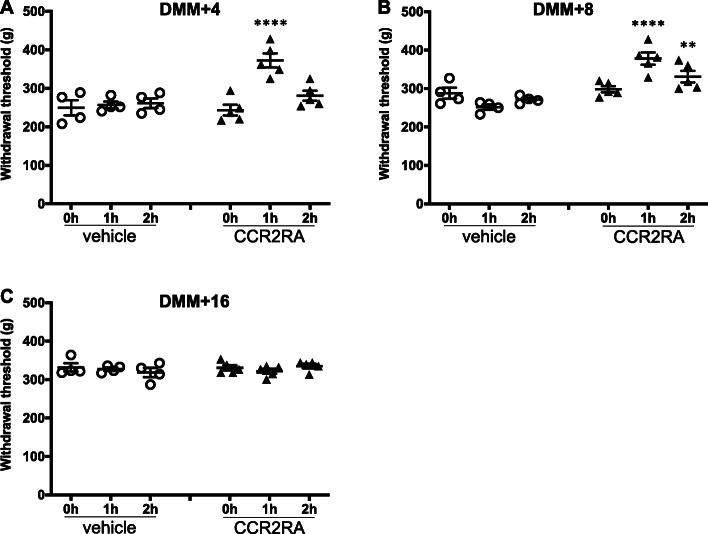
CCR2RA delivered systemically at different time points after DMM surgery. **a** 4, **b** 8, and **c** 16 weeks after DMM surgery, CCR2RA (RS504393, Tocris: 5 mg/kg, i.p., *n* = 5) or vehicle control (100% DMSO_4_, i.p., *n* = 4) was systemically administered and knee hyperalgesia was measured before injection, 1 h after, and 2 h after injection. **p < 0.01, ****p < 0.0001 vs vehicle. Repeated measures two-way ANOVA with Bonferroni’s multiple comparisons test to compare CCR2-RA to vehicle at each time point. Mean ± SEM

Confirming our previous findings [28, 37], WT mice (*n* = 5) developed knee hyperalgesia 2 weeks after DMM surgery, which gradually recovered by week 16 (Fig. 1a; *p* < 0.0001 for 4–12 weeks after surgery compared to pre-surgery; *p* = 0.0041 for 16 weeks after surgery compared to pre-surgery). In contrast, *Ccr2* null mice (*n* = 5) did not develop hyperalgesia up to 16 weeks after DMM (Fig. 1b). These findings suggest that CCR2 signaling is essential for development of knee hyperalgesia after DMM surgery. In order to assess the role of CCR2 signaling at different stages of the model, we tested the effect of systemically administered CCR2RA, which blocks CCR2 signaling, on established knee hyperalgesia at different time points after DMM. DMM surgery was performed in 10-week old WT mice (*n* = 9). CCR2RA (RS504393, 5 mg/kg) (*n* = 5 mice) or vehicle control (DMSO_4_) (*n* = 4 mice) was injected i.p., 4, 8, or 16 weeks after surgery, and knee hyperalgesia was assessed. Four weeks after DMM surgery, hyperalgesia in the operated knee was reversed 1 h after CCR2RA injection (*p* < 0.0001 vs. vehicle) (Fig. 2a); 8 weeks after surgery, CCR2RA reversed hyperalgesia 1 and 2 h after administration (*p* < 0.0001 at 1 h and *p* = 0.008 at 2 h vs. vehicle) (Fig. 2b). Sixteen weeks after DMM surgery, there was no difference between the vehicle control and treatment groups (Fig. 2c) (*p* > 0.99 at 1 h and *p* = 0.46 at 2 h vs. vehicle).

### CCL2 injection into the knee joint cavity directly excites sensory afferents and results in transient knee hyperalgesia in WT but not in *Ccr2* null mice

**Fig. 3:**
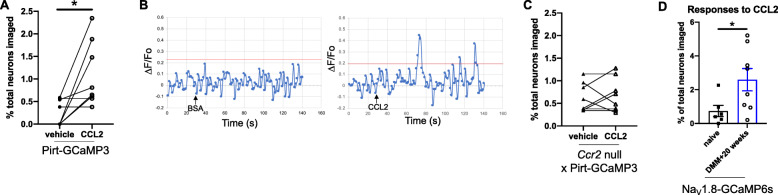
In vivo calcium imaging testing neuronal responses to i.a. injection of vehicle (7 μL, 0.1% BSA in PBS) or CCL2 (7 μL, #MJE00, R&D systems, 700 ng). **a** Percentage of neurons that responded to vehicle or CCL2 in naïve Pirt-GCaMP3 mice. Each dot = one mouse. *n* = 8; **b** Example ∆F/Fo plots are shown to depict how responses were determined. This neuron responded to CCL2 but not to BSA in a naïve Pirt-GCaMP3 mouse (this mouse had 0% vehicle responses and 1.5% CCL2 responses); Red line indicates the threshold for response = 4× standard deviation of the first 15 frames; Each dot indicates an imaging frame—the connecting line is included for ease of viewing peaks. **c** Percentage of neurons that responded to vehicle or CCL2 in naïve *Ccr2* null × Pirt-GCaMP3 mice. *n* = 8; Mean ± SEM. Paired two-tailed *t* test, *p < 0.05. **d** Percentage of neurons that responded to CCL2 in naïve (*n* = 6) or DMM + 20 week (*n* = 8) Na_V_1.8 cre-GCaMP6s loxp mice. Unpaired two-tailed *t* test. *p < 0.05

In order to assess the direct contribution of neuronal CCR2 to development of knee hyperalgesia, we assessed rapid-onset effects of i.a. injection of CCL2 into the knee joint space. We have previously shown that approximately 15% of all sensory neurons located in the L4-DRG innervate the knee joint [29]. Here, we assessed whether i.a. injection of CCL2 into a healthy mouse knee can directly excite those sensory neurons, through in vivo calcium imaging of the L4-DRG. We found that injecting vehicle into the knee cavity of naïve Pirt-GCaMP3 mice elicited a response in a very small number of neurons (8 mice, 0.26 ± 0.1% of L4-DRG neurons), while injecting CCL2 elicited responses in an increased percentage of sensory neurons (1.1 ± 0.25% of neurons) (Fig. 3a; *p* = 0.02). Figure 3b represents an example response, showing an increase in transient [Ca^2+^]_i_ following injection of CCL2. In contrast, *Ccr2* null × Pirt-GCaMP3 mice showed a similar percentage of neuronal responses to vehicle and CCL2 injections (Fig. 3c; *n* = 8; vehicle: 0.63 ± 0.11%; CCL2: 0.68 ± 0.13%, *p* = 0.68).

In order to assess whether responses to CCL2 change after DMM surgery, we used Na_V_1.8 cre × GCaMP6s loxp mice to measure CCL2 elicited responses specifically in nociceptors in naïve mice and in mice 20 weeks after DMM surgery. We found that a similar percentage of neurons responded to i.a. CCL2 in naïve Na_V_1.8-GCaMP6s mice (0.74 ± 0.33% of neurons) compared to naïve Pirt-GCaMP3 mice. In addition, after DMM surgery, the percentage of neurons responding to CCL2 increased compared to naïve mice (2.6 ± 0.66% of neurons; *p* = 0.04) (Fig. 3d).

**Fig. 4:**
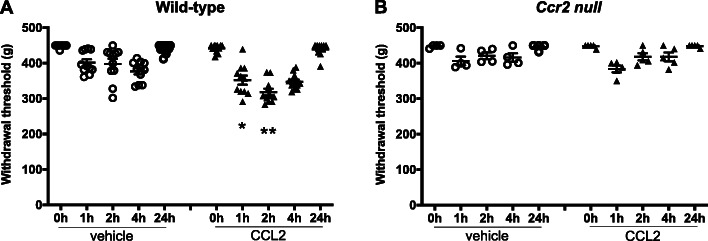
CCL2 (#MJE00, R&D systems, 500 ng (5 μL) i.a.) or vehicle (5 μL of 0.1% BSA in PBS) injected intra-articularly into **a** naïve wild-type and **b** naïve *Ccr2* null mice. Knee hyperalgesia was measured before injection, and 1, 2, 4, and 24 h after injection. *n* = 11 vehicle, *n* = 11 CCL2 for wild-type; *n* = 4 vehicle, *n* = 5 CCL2 for *Ccr2* null mice. *p < 0.05, **p < 0.01 vs vehicle at the same time point. Repeated measures two-way ANOVA with Bonferroni’s multiple comparisons test to compare CCL2 to vehicle at each time point. Mean ± SEM

The ability of locally administered CCL2 to directly activate sensory neurons upon i.a. injection suggests that this chemokine may be able to cause rapid-onset knee hyperalgesia upon local injection. We tested this experimentally by injecting CCL2 into the knees of naive WT mice. CCL2 (100 ng/μL in 5 μl) or vehicle (5 μL PBS + 0.1% BSA) was injected i.a. into the right knee of 9-week-old male naïve WT mice (11 mice/group). Knee hyperalgesia was assessed right before injection and then 1, 2, and 4 h after administration of CCL2, and again 20 h later. Injecting CCL2 into the knee cavity resulted in knee hyperalgesia 1 and 2 h later, compared to vehicle control (Fig. 4a, *p* = 0.03 at 1 h; *p* = 0.001 at 2 h; CCL2 vs. vehicle). The withdrawal threshold recovered to baseline by 24 h after CCL2 injection (Fig. 4a). The rapid onset of knee hyperalgesia after i.a. injection suggests this is a direct, neuronally mediated effect. In order to establish that this was a specific CCR2 signaling mediated effect, we repeated the same experiment in 9-week-old naïve male *Ccr2* null mice. We found that vehicle (*n* = 4 mice) or 500 ng CCL2 (*n* = 5 mice) injection were not capable of triggering knee hyperalgesia in *Ccr2* null mice at any time up to 24 h (Fig. 4b; *p* = 0.39 at 1 h, CCL2 vs. vehicle), further suggesting that local CCL2-CCR2 signaling mediates knee hyperalgesia.

### Intra-articular administration of CCR2RA reverses knee hyperalgesia after DMM

**Fig. 5:**
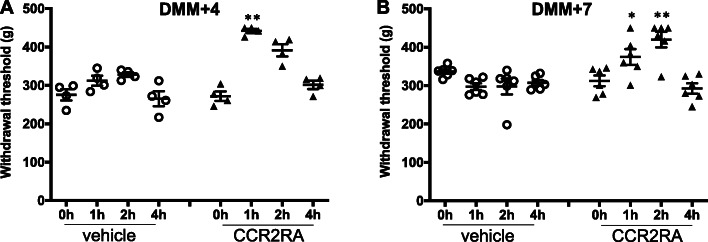
CCR2RA delivered intra-articularly at different time points after DMM surgery. **a** Four and **b** 7 weeks after DMM surgery, CCR2RA (BMS CCR2 22, R&D systems: 0.5 mg/kg, i.a.) or vehicle control (50% EtOH, i.a.) was intra-articularly administered and knee hyperalgesia was measured before injection, and 1, 2, and 4 h after injection. (*n* = 4 vehicle, *n* = 4 CCR2RA at DMM + 4 weeks) (*n* = 6 vehicle, *n* = 6 CCR2RA at DMM + 7 weeks) *p < 0.05, **p < 0.01 vs vehicle. Repeated measures two-way ANOVA with Bonferroni’s multiple comparisons test to compare CCR2RA to vehicle at each time point. Mean ± SEM

In order to further establish whether CCR2 signaling in the joint acts locally to establish knee hyperalgesia in the context of experimental OA, we decided to test the effect of i.a. administered CCR2RA in DMM mice with established knee hyperalgesia. DMM surgery was performed in 10-week old WT mice (*n* = 20 mice), and CCR2RA (BMS CCR2 22, 0.5 mg/kg) or vehicle control (50% ethanol) was injected i.a. into the knee joint, 4 weeks (*n* = 4 vehicle and *n* = 4 CCR2RA) and 7 weeks (*n* = 6 vehicle and *n* = 6 CCR2RA) after surgery. Four weeks after DMM, i.a. injection of CCR2RA, but not vehicle, reversed established knee hyperalgesia by 1 h after injection (*p* = 0.0029 vs. vehicle) (Fig. 5a). Knee hyperalgesia returned 4 h after injection (Fig. 5a). Seven weeks after DMM surgery, the maximum effect of CCR2RA was observed 2 h after injection (*p* = 0.007 vs. vehicle) and knee hyperalgesia returned by 4 h after the injection (Fig. 5b).

### CCL2 protein is elevated in whole joint extracts 4 weeks after DMM in WT and in *Ccr2* null mice

Because the previous results suggest that i.a. injection of CCR2RA blocks the effects of locally produced CCL2 in the knee, we measured total CCL2 protein levels in the knees of WT mice, 4 and 8 weeks after sham or DMM surgery in the right knees. Four weeks after DMM, CCL2 levels were elevated compared to naïve or sham-operated mice (*p* = 0.018 vs. naïve; *p* = 0.048 vs. sham), but levels were back to baseline by week 8 (Supp Fig. 1). We also performed DMM surgery in *Ccr2* null mice and measured CCL2 levels in whole knee joint extracts. Here, we found that CCL2 levels were also elevated, again peaking 4 weeks after DMM (*p* = 0.013 vs. naive) (Supp Fig. 1).

### CCR2 is expressed by intra-articular nociceptors

**Fig. 6:**
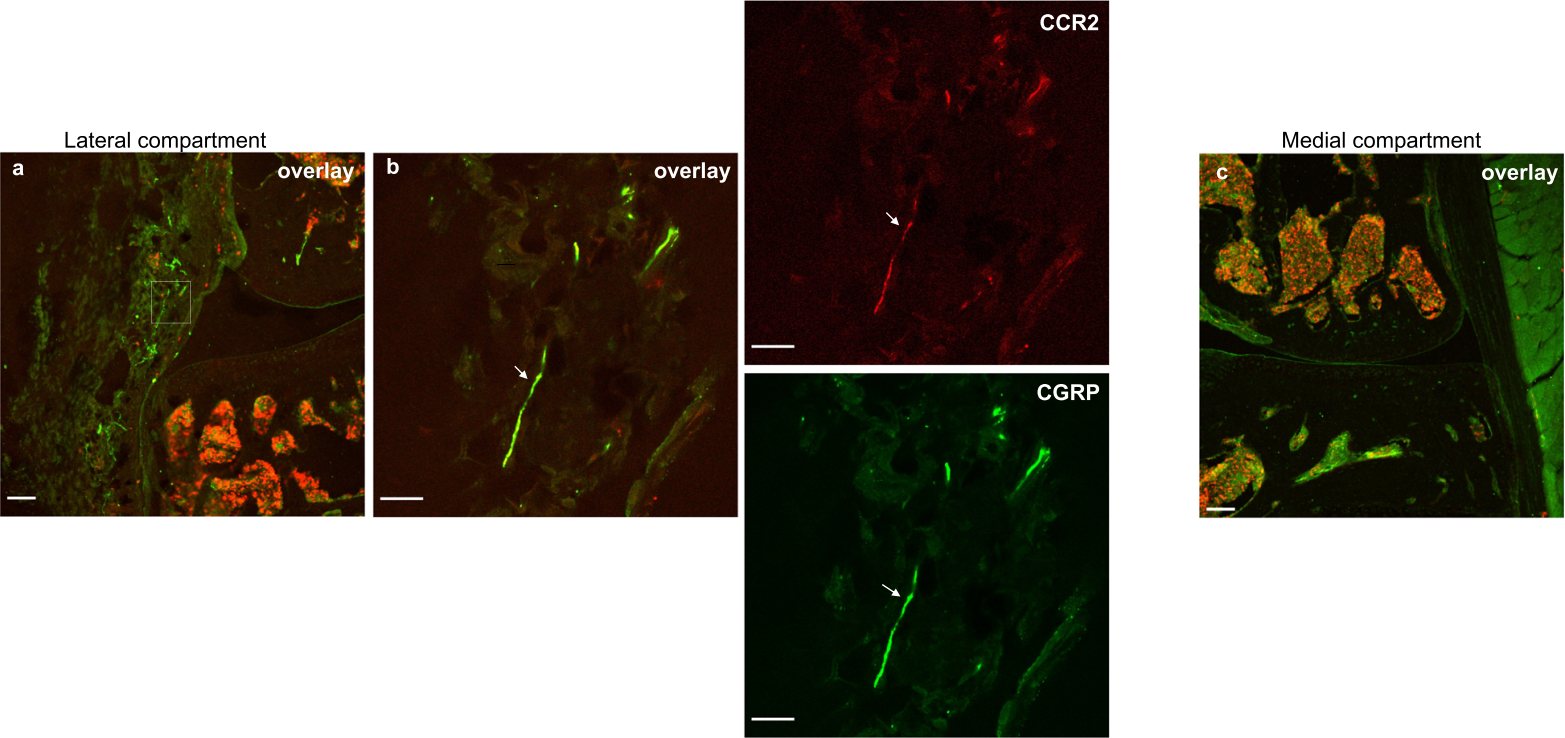
Representative confocal images of the right knee of a 10-week-old male naïve *Ccr2*^RFP^ mouse, immunostained with CGRP. **a** CGRP/CCR2 overlay of the lateral compartment of the knee; **b** magnified white inset of **a** showing CCR2, CGRP signals and the overlay of both images. White arrows indicate an example of an overlap between CGRP and CCR2 signals (CCR2 receptors present on CGRP+ nerve fiber). **c** CGRP/CCR2 overlay of the medial compartment of the knee, an area where we do not see a high amount of innervation in naïve mice. Scale bar for *a*, *c* = 100 μm. Scale bar for *b* = 25 μm

In order to visualize the location of CCR2 on intra-articular afferents, *Ccr2*^RFP^ mice and immunohistochemical staining for the nociceptor marker, calcitonin gene-related peptide (CGRP), or the pan-neuronal marker, protein gene product 9.5 (PGP9.5), were used. Sections from 10-week-old male naïve *Ccr2*^RFP^ mice (*n* = 3) showed overlap between CGRP staining and CCR2-RFP signal in the lateral synovium (Fig. 6 a, b), an area that we have previously shown to have dense nociceptive innervation in naïve mice [33]. In addition, in another set of naïve *Ccr2*^RFP^ mice (*n* = 4), we observed some overlap between CCR2 and PGP9.5 signals (Supp. Fig. 2 A-D). No CCR2 signal was seen in the medial synovium (Fig. 6c)—we have previously also demonstrated a lack of nociceptors in this compartment in young naïve mice [33].

**Fig. 7:**
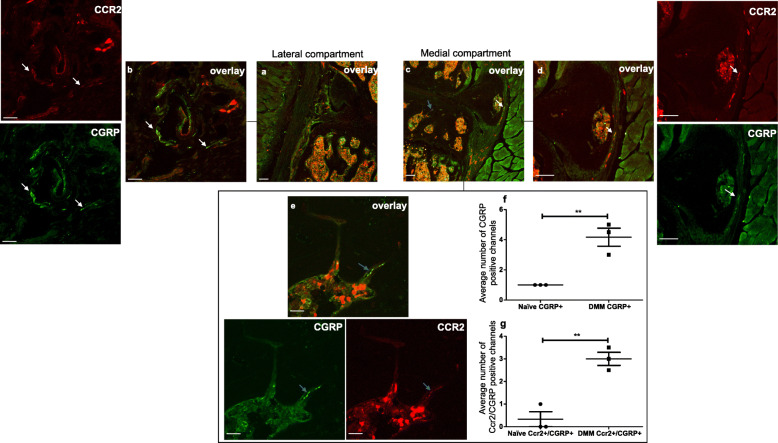
Representative confocal images of the right knee of a *Ccr2*^*RFP*^ mouse 8 weeks after DMM surgery, immunostained with CGRP. **a** CGRP/CCR2 overlay of the lateral compartment of the knee; **b** magnified image of **a** showing CCR2, CGRP signals and the overlay of both images. White arrows indicate an example of overlap between CGRP and CCR2 signals in the lateral synovium (CCR2 receptors present on CGRP+ nerve fibers). **c** CGRP/CCR2 overlay of the medial compartment of the knee; **d**, **e** magnified of **c** showing CCR2, CGRP signals and the overlays of both images. **c**, **d** White arrows indicate an example of an overlap between CGRP and CCR2 signal in the medial synovium (CCR2 receptors present on CGRP+ nerve fibers). **c**, **e** Blue arrows point to a CGRP/CCR2-positive channel in the medial subchondral bone. **f**, **g** Quantification of the CGRP+ and CCR2+/CGRP+ channels in the subchondral bone of naïve and DMM knees. Unpaired two-tailed *t* test. Scale bar for *a*, *c*, *d* = 100 μm. Scale bar for *b*, *e* = 25 μm. **p < 0.01. Mean ± SEM

Because we have shown that new sensory nerves develop in the medial compartment after DMM surgery [33], we also examined *Ccr2*^RFP^ mice 8 weeks after DMM. We found that these mice still had CGRP+ innervation in the lateral synovium, similar to naïve mice, with a subset of fibers showing overlay with CCR2 (Fig. 7 a, b, Supp. Fig. 3). In addition, we found that these mice developed increased numbers of CGRP+ osteochondral channels in the medial compartment compared to naïve controls, as has been reported in rat and human OA [38] (Fig. 7 c, f, Supp. Fig. 3). In addition, a subset of the CGRP+ nerves in these channels also showed overlap with CCR2-RFP signal (Fig. 7e, Supp. Fig. 3), and the number of double-positive CCR2+/CGRP+ channels increased after DMM (Fig. 7g). We also observed some overlap between CGRP and CCR2-RFP in the medial synovium (Fig. 7d, Supp. Fig. 3), in the area where we have seen Na_V_1.8+ nerves appear after DMM surgery previously [33].

## Discussion

In this study, we aimed to assess whether CCR2 expressed by intra-articular afferents can respond to CCL2, and how this may relate to knee hyperalgesia. The findings suggest that CCL2-CCR2 signaling locally in the joint contributes to knee hyperalgesia in experimental OA, and the effect is in part mediated through direct stimulation of CCR2 expressed by intra-articular sensory afferents. These findings are of interest for several reasons, discussed below.

First, many studies have reported that levels of chemokines, including CCL2 as well as other CCR2 ligands, CCL7 and CCL8, are elevated in osteoarthritic compared to healthy joints [39, 40]. CCL2 levels are increased in the synovial fluid of human OA knees, and this has been correlated with symptoms [41, 42]. A recent study reported that CCL2 was one of a panel of 6 synovial fluid biomarkers related to synovial inflammation—specifically to macrophage activation—in OA, as well as to radiographic and symptom severity [43]. Interestingly, several independent studies have reported that, after joint trauma, CCL2 levels in the synovial fluid rapidly increase [44], and levels correlated with pain [44–46]. CCL2 is also increased in the joints in experimental models of OA. For example, after DMM surgery, *Ccl2* is rapidly induced in the joint [15]. In a rat surgical model, *Ccl2* was elevated in the cartilage early after surgery [47]. Here, we report increased CCL2 protein in whole knee joint extracts 4 weeks after DMM but not sham surgery. Therefore, this chemokine is a good candidate mediator of the knee hyperalgesia that occurs early on after DMM surgery [28]. Indeed, we found that *Ccr2* null mice were protected from developing knee hyperalgesia after DMM surgery. Furthermore, locally injected CCR2RA reversed ongoing knee hyperalgesia at 4 weeks. CCR2RA was also efficacious in reversing knee hyperalgesia when injected at later stages, i.e., 7 weeks after DMM, when CCL2 levels in the joint are declining. There are several explanations for this observation. For one, there may still be sufficient CCL2 present in specific joint tissues to activate local nociceptors, since many cells can produce this chemokine, including chondrocytes and synoviocytes [39]. Moreover, DRG neurons can also release CCL2 and they increase their production of this chemokine in pain states—for example, we have previously shown that DRG neurons produce more CCL2 protein 8 weeks after DMM [13]. This indicates the potential existence of a positive autocrine CCR2-mediated feedback loop that operates in joint pain. Finally, other CCR2 ligands may be present in the joint at the 7–8 week time point. Indeed, it has been shown by IHC that CCL12 is upregulated in the joint 4, 8, and 12 weeks after DMM surgery [14].

A second observation of interest is that the involvement of CCR2 signaling appears to operate at the level of the joint, since i.a. injection of CCL2 caused rapid-onset transient hyperalgesia in WT but not in *Ccr2* null mice. Furthermore, this effect is likely mediated through CCR2 expressed by i.a. neurons, as suggested by in vivo calcium imaging experiments showing that neurons in the L4-DRG of naïve WT—but not *Ccr2* null—mice respond to CCL2 injected into the knee cavity. These experiments were modeled on classical experiments describing that i.a. injection of cytokines such as TNF⍺, IL-1, IL-6, and IL-17 can rapidly activate nerves as demonstrated by in vivo electrophysiology [48, 49]. This was further strengthened by our finding that CCR2 and CGRP, as well as CCR2 and PGP9.5, co-localized in the knee. Reports of expression of specific receptors on i.a. nerves are exceedingly rare [50], and this has not been shown for CCR2. More commonly, i.a. receptor expression is inferred by actions of drugs injected into the knee joint. Description of receptor expression by sensory neurons is usually focused on the cell bodies in the DRGs, as has been shown for CCR2. In addition, a variety of receptors have been shown to be expressed by afferents in the skin [51]. Hopefully, the availability of reporter mice will enable future studies to describe the detailed localization of receptors on peripheral nerve terminals innervating the joint in the course of OA.

We and others have previously shown a role for CCR2 signaling in pain associated with OA joint damage in the DMM model [13]. Specifically, we previously reported that *Ccr2* null mice were protected from persistent pain behaviors in this model [13]. Furthermore, both *Ccr2* null and *Ccl2* null mice had a delayed onset of weightbearing deficits after DMM surgery [15]. This protection against chronic pain occurred despite *Ccr2* null mice developing comparable joint damage as WT mice as well as comparable early upregulation of other inflammatory markers in the knee joint [15]. CCR2RA administered 10 weeks after DMM alleviated locomotor deficits associated with this model [13], and in one study, chronic systemic administration of CCR2RA during the first 4 weeks after DMM inhibited development of weightbearing deficits [14]. Interestingly, this analgesic effect was sustained even at later stages, even when the drug was only administered the first 4 weeks after surgery [14]. The current study is the first to examine the role of this pathway in the development of knee hyperalgesia, which is an indicator of local joint pain and largely an early-stage pain behavior in this model. The findings that locally injected CCR2RA can reverse this behavior 4–7 weeks after surgery, suggest that this pathway may constitute an attractive analgesic target for early stages of disease, for example immediately after joint injury.

Finally, we have recently reported that the nociceptive innervation of the knee is profoundly altered after DMM surgery, both anatomically with the appearance of new nerve endings in the medial compartment [33], and functionally with increased numbers of sensory neurons responding to an applied mechanical force [29]. Here, we provide evidence that a subset of these new nerves also express CCR2 and are more responsive to CCL2 injected into the knee joint, suggesting that CCL2-CCR2 signaling is contributing to this neuroplasticity in OA.

## Conclusions

We have previously demonstrated that CCL2-CCR2 signaling is an important mediator of pain during the development of experimental OA induced by surgical DMM [13]. In this context, CCR2 signaling may contribute to pain-related responses through recruiting macrophages to the injured joint and to the DRG, as well as through direct neuronal activation. The latter is associated with the expression of both CCL2 and CCR2 by nociceptors in the DRG and resulting CCR2-mediated nociceptor excitation. In order to be able to intervene in this process effectively, it is important to elucidate whether algogenic signaling takes place within the knee joint or at the level of the DRG, through intersomatic CCR2 signaling [52]. These two sites are anatomically distinct due to the morphology of DRG neurons. The results of the experiments discussed above now make it clear that, at least during the initial phase after DMM surgery, CCR2 mediated signaling that occurs solely in the knee joint is sufficient to produce DMM-associated hyperalgesia, suggesting that blocking CCR2 responses at peripheral terminals is sufficient to produce analgesic effects in OA. It will be interesting in future experiments to investigate whether chronic administration of CCR2RA is effective in delaying onset of knee hyperalgesia. In addition, reports of expression of specific receptors on intra-articular nerves are exceedingly rare—translationally, it will be important to identify what other druggable receptors are expressed by intra-articular afferents in OA. These observations will help to translate these results into the clinical arena. All our findings point toward a temporally tightly regulated role of this pathway in controlling pain in OA—as every study attempting to define this role has found [5].

## Supplementary Information

**Additional file 1: Supp Fig. 1**. CCL2 protein expression in whole knee joint extracts collected from wild-type (wt) and *Ccr2* null mice at different time points after sham or DMM surgery. (*n* = 4 mice wt naïve 14 weeks old; *n* = 6 wt sham + 4; *n* = 11 wt dmm + 4; n = 4 wt naïve 18 weeks old; *n* = 5 wt sham+ 8; n = 11 wt dmm + 8; n = 6 *Ccr2* null naïve 10 weeks old; *n* = 8 *Ccr2* null DMM + 4; n = 5 *Ccr2* null DMM + 8) *p < 0.05, **p < 0.01 vs vehicle. One-way ANOVA with Tukey’s multiple comparisons test to compare treatment groups at each time point. Mean ± SEM. **Supp. Fig. 2.** Representative confocal images of the right knee of a 10-week old male naïve Ccr2^RFP^ mouse, immunostained with PGP9.5. (A,B) Two different magnifications of a representative frontal section demonstrating PGP9.5/CCR2 overlays within the lateral synovium of the knee; (C,D) magnified white insets of (B) showing CCR2, PGP9.5 signals and the overlays of both images. White arrows indicate the exact areas of the overlap between PGP9.5 and CCR2 signals (CCR2 receptors present on PGP9.5+ nerve fibers). Scale bar for A,B = 100 μm. Scale bar for the rest of images = 25 μm. **Supp. Fig. 3**. Representative confocal images of the right knee of Ccr2^RFP^ mouse 8 weeks after DMM surgery from a different mouse than shown in Fig. 7, immunostained with CGRP. (a, b) CGRP/CCR2 overlay of the lateral and medial compartments of the knee respectively; (c) magnified of (b) showing CCR2, CGRP signals and the overlay of both images. Blue arrows point to a CGRP+/CCR2+ channel in the medial subchondral bone. (d) magnified of (a) showing CCR2, CGRP signals and the overlays of both images. White arrows point to CGRP+/CCR2+ signal in the lateral synovium. Scale bar for a,b = 100 μm. Scale bar for c,d = 25 μm.

## Data Availability

All data generated or analyzed during this study are included in this published article [and its supplementary information files].
