# Supplementary material for: The role of intra-articular neuronal CCR2 receptors in knee joint pain associated with experimental osteoarthritis in mice
Source: Arthritis Res Ther. 2021 Apr 7;23:103. doi: 10.1186/s13075-021-02486-y (PMC8025346; doi:10.1186/s13075-021-02486-y)
Supplement: Supplementary file 1 — Additional file 1: Supp Fig. 1. CCL2 protein expression in whole knee joint extracts collected from wild-type (wt) and Ccr2 null mice at different time points after sham or DMM surgery. (n = 4 mice wt naïve 14 weeks old; n = 6 wt sham + 4; n = 11 wt dmm + 4; n = 4 wt naïve 18 weeks old; n = 5 wt sham+ 8; n = 11 wt dmm + 8; n = 6 Ccr2 null naïve 10 weeks old; n = 8 Ccr2 null DMM + 4; n = 5 Ccr2 null DMM + 8) *p < 0.05, **p < 0.01 vs vehicle. One-way ANOVA with Tukey’s multiple comparisons test to compare treatment groups at each time point. Mean ± SEM. Supp. Fig. 2. Representative confocal images of the right knee of a 10-week old male naïve Ccr2RFP mouse, immunostained with PGP9.5. (A,B) Two different magnifications of a representative frontal section demonstrating PGP9.5/CCR2 overlays within the lateral synovium of the knee; (C,D) magnified white insets of (B) showing CCR2, PGP9.5 signals and the overlays of both images. White arrows indicate the exact areas of the overlap between PGP9.5 and CCR2 signals (CCR2 receptors present on PGP9.5+ nerve fibers). Scale bar for A,B = 100 μm. Scale bar for the rest of images = 25 μm. Supp. Fig. 3. Representative confocal images of the right knee of Ccr2RFP mouse 8 weeks after DMM surgery from a different mouse than shown in Fig. 7, immunostained with CGRP. (a, b) CGRP/CCR2 overlay of the lateral and medial compartments of the knee respectively; (c) magnified of (b) showing CCR2, CGRP signals and the overlay of both images. Blue arrows point to a CGRP+/CCR2+ channel in the medial subchondral bone. (d) magnified of (a) showing CCR2, CGRP signals and the overlays of both images. White arrows point to CGRP+/CCR2+ signal in the lateral synovium. Scale bar for a,b = 100 μm. Scale bar for c,d = 25 μm. [file 13075_2021_2486_MOESM1_ESM.pdf]

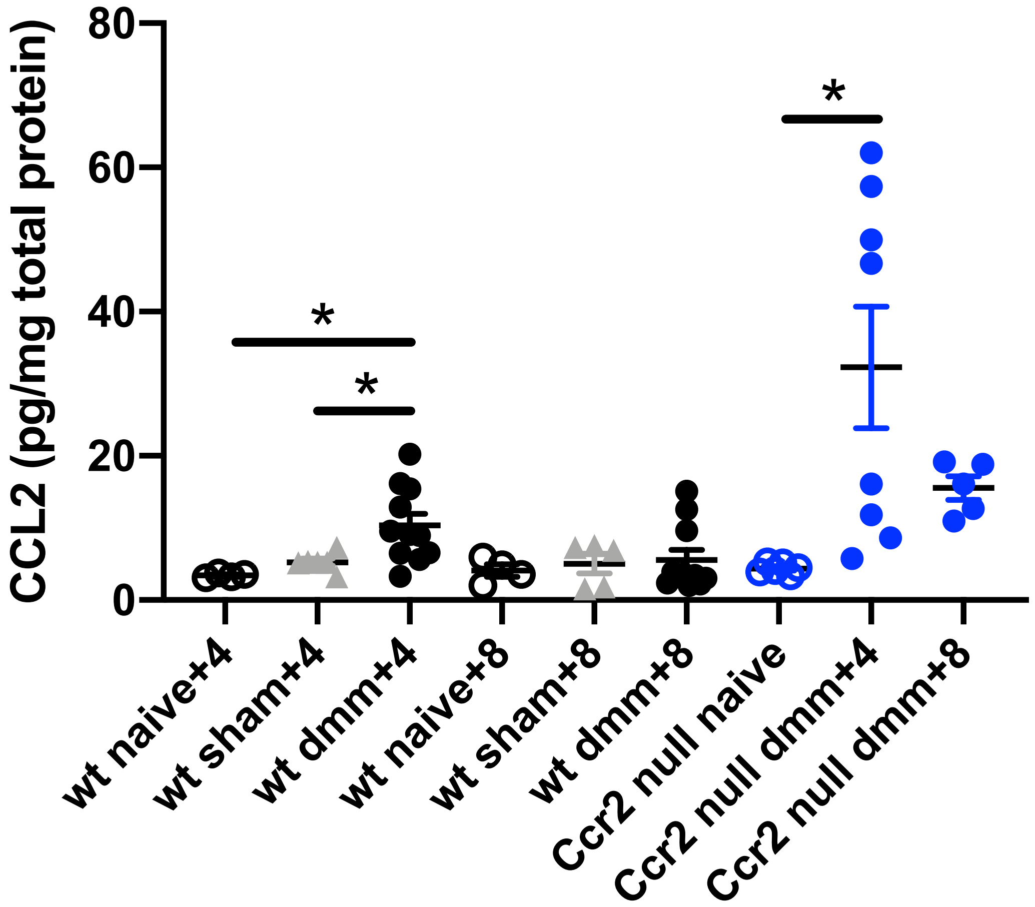

Supp Fig. 1. CCL2 protein expression in whole knee joint extracts collected from wild-type (wt) and *Ccr2* null mice at different time points after sham or DMM surgery. (n=4 mice wt naïve 14 weeks old; n=6 wt sham +4; n=11 wt dmm+4; n=4 wt naïve 18 weeks old; n=5 wt sham+8; n=11 wt dmm+8; n=6 *Ccr2* null naïve 10 weeks old; n=8 *Ccr2* null DMM+4; n=5 *Ccr2* null DMM+8) *p<0.05, **p<0.01 vs vehicle. One-way ANOVA with Tukey's multiple comparisons test to compare treatment groups at each time point. Mean±SEM.

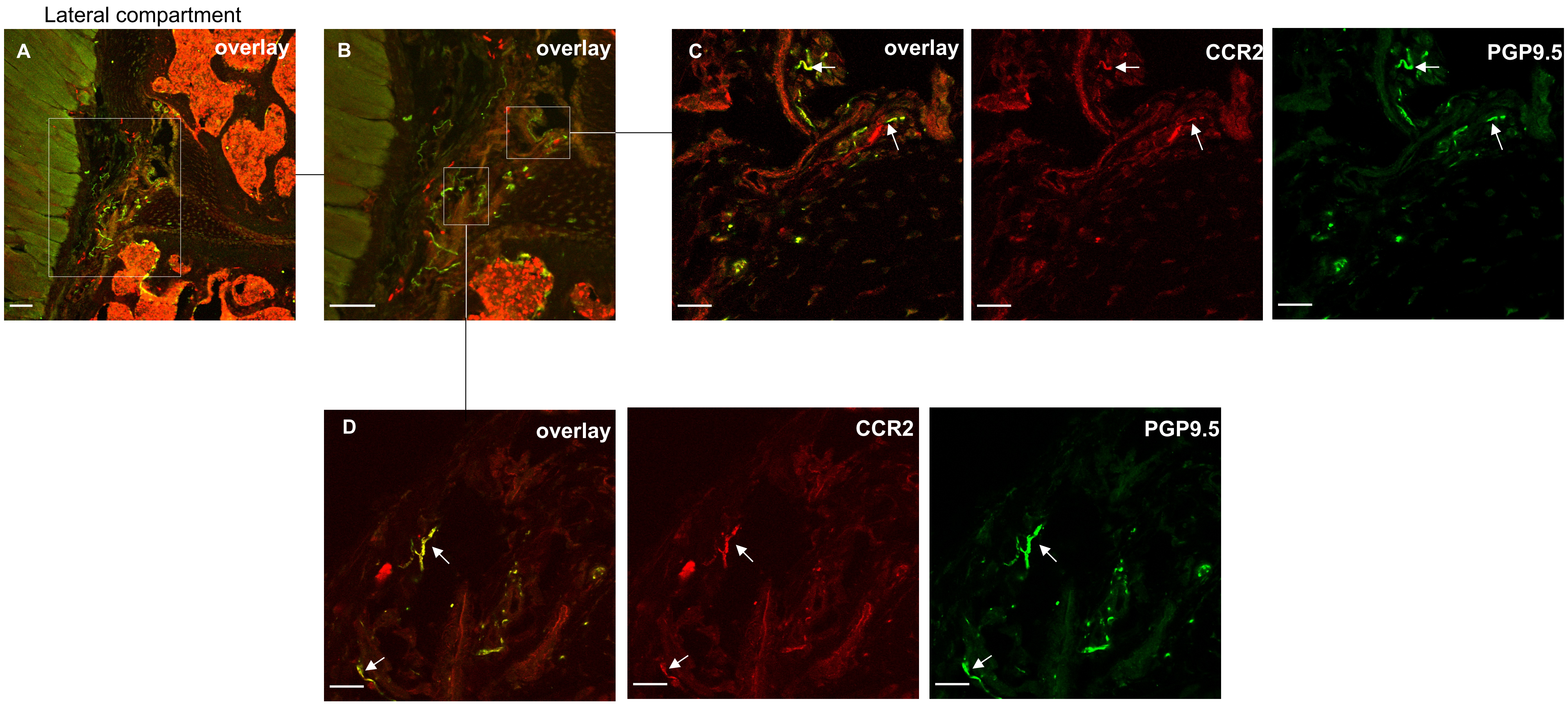

Supp. Fig. 2: Representative confocal images of the right knee of a 10-week old male naïve Ccr2^{RFP} mouse, immunostained with PGP9.5. **(A,B)** Two different magnifications of a representative frontal section demonstrating PGP9.5/CCR2 overlays within the lateral synovium of the knee; **(C,D)** magnified white insets of **(B)** showing CCR2, PGP9.5 signals and the overlays of both images. White arrows indicate the exact areas of the overlap between PGP9.5 and CCR2 signals (CCR2 receptors present on PGP9.5+ nerve fibers). Scale bar for **A,B** = 100 µm. Scale bar for the rest of images = 25 µm.

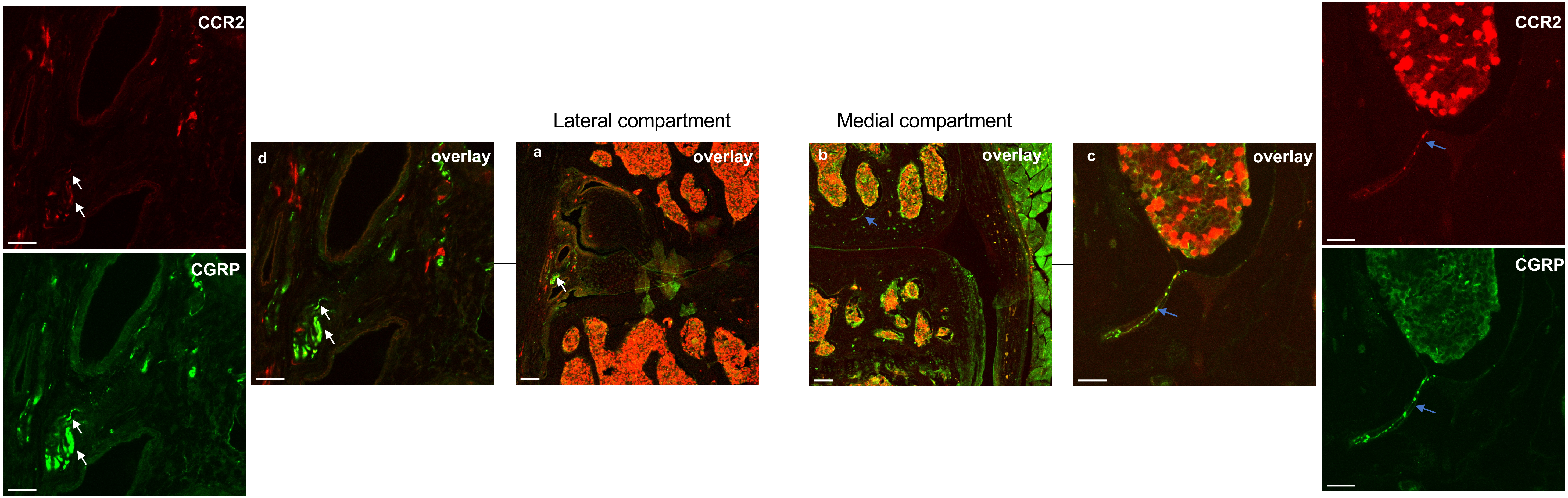

Suppl Figure 3: Representative confocal images of the right knee of Ccr2^{RFP} mouse 8 weeks after DMM surgery from a different mouse than shown in Fig. 7, immunostained with CGRP. (a, b) CGRP/CCR2 overlay of the lateral and medial compartments of the knee respectively; (c) magnified of (b) showing CCR2, CGRP signals and the overlay of both images. Blue arrows point to a CGRP+/CCR2+ channel in the medial subchondral bone. (d) magnified of (a) showing CCR2, CGRP signals and the overlays of both images. White arrows point to CGRP+/CCR2+ signal in the lateral synovium. Scale bar for a,b = 100 µm. Scale bar for c,d = 25 µm.
